# The Geography of Disease

**Published:** 1904-03

**Authors:** 


**The Geography of Disease.** By FRANK CLEMOW, M.D. Pp. xiv., 624. Cambridge: The University Press. 1903.

This may be a useful book of reference to medical men interested in tropical diseases, but we have been unable to discover many noteworthy facts not mentioned in good textbooks on medicine. It is interesting, however, to notice how widespread is a disease like rheumatism, and that it occurs in places as different as Greenland, Senegal, and South Africa. That chronic rheumatic affections occurred in Egypt in ancient days is well known from examination of the bones of mummies. Apparently in North Africa these chronic forms are still common, while acute articular rheumatism is rare. Rickets is a disease that we might perhaps suppose to be a disease of civilisation; but in some of the Pacific Islands it is said to be common, and in the New Hebrides the greater part of the children are said to be either rickety or scrofulous. The disease is said to be very rare in India, not uncommon in Burmah, but unknown in Siam. Apparently no very definite deductions in connection with its ætiology can be made from its distribution. Neither race nor climate seems to have a very marked effect. The writer, however, says "it would almost seem that a plentiful supply of pure air is of greater importance in preventing (and in curing) this malady than the nature of the diet."

